# Semantic systems are mentalistically activated for and by social partners

**DOI:** 10.1038/s41598-022-08306-w

**Published:** 2022-03-22

**Authors:** Bálint Forgács, Judit Gervain, Eugenio Parise, György Gergely, Lívia Priyanka Elek, Zsuzsanna Üllei-Kovács, Ildikó Király

**Affiliations:** 1 Department of Cognitive Psychology, Institute of Psychology, ELTE Eötvös Loránd University, Izabella utca 46, 1064 Budapest, Hungary; 2 Dipartimento di Psicologia dello Sviluppo e della Socializzazione - DPSS, Università Padua, 35131 Padua, Italy; 3 Integrative Neuroscience and Cognition Center, Université de Paris, 75006 Paris, France; 4 CIMeC - Center for Mind/Brain Sciences, University of Trento, Trento, Italy; 5 Department of Psychology, Lancaster University, Lancaster, LA1 4YF UK; 6 Cognitive Development Center (CDC), Department of Cognitive Science, Central European University (CEU), 1051 Budapest, Hungary

**Keywords:** Psychology, Cognitive neuroscience, Social neuroscience

## Abstract

A recently discovered electrophysiological response, the social N400, suggests that we use our language system to track how social partners comprehend language. Listeners show an increased N400 response, when themselves not, only a communicative partner experiences a semantic incongruity. Does the N400 reflect purely semantic or mentalistic computations as well? Do we attribute language comprehension to communicative partners using our semantic systems? In five electrophysiological experiments we identified two subcomponents of the social N400. First, we manipulated the presence-absence of an Observer during object naming: the semantic memory system was activated by the presence of a social partner in addition to semantic predictions for the self. Next, we induced a false belief—and a consequent miscomprehension—in the Observer. Participants showed the social N400, over and above the social presence effect, to labels that were incongruent for the Observer, even though they were congruent for them. This effect appeared only if participants received explicit instructions to track the comprehension of the Observer. These findings suggest that the semantic systems of the brain are not merely sensitive to social information and contribute to the attribution of comprehension, but they appear to be mentalistic in nature.

## Introduction

Mentalization, attributing intentions, beliefs and desires to social partners, may be an integral part of language use and comprehension. Human communication has been proposed to be inferential in nature^1,2^, where linguistic meaning is established based on semantic content in combination with communicative intentions. In the present paper we set out to investigate the neurocognitive mechanisms involved in the mentalistic inferential processes during linguistic communication.

In a series of electroencephalography (EEG) experiments we aimed to find out if the so-called social N400 effect is semantic in nature or mentalistic as well. The typical N400 is an event-related potential (ERP) component that can be evoked by semantic incongruities (“He spread the warm bread with socks”)^3^. Even though it can be elicited by semantic violations, it responds to various semantic factors in a graded fashion and appears whenever semantic predictions are not fully met^4^. Consequently, the N400 is thought to index a semantic memory retrieval effort and best conceptualized as always being evoked, but reduced to the extent that semantic expectations are fulfilled^5^. Some alternative accounts suggest that the N400 may reflect semantic integration^6^, however, this interpretation appears to be contradicted by observations that the effect can be elicited by manipulating whether an article is definite or not^7,8^, and an exhaustive review also concluded that the memory retrieval account appears to provide a more accurate description of the available data^9^. Thus, here we will assume the semantic memory retrieval account, noting that from the perspective of the present paper the actual mechanism involved is not of primary concern.

Recent results show that the same N400 response is also triggered when not the self but a social partner experiences semantic incongruities, which is termed the social N400 effect^10–12^. Participants produced an N400 response in the presence of a confederate, for whom a visually presented target sentence was incongruent even though it was rendered congruent for participants by an extra context sentence presented in headphones. The effect disappears under cognitive pressure or in lack of instructions to follow the comprehension of the confederate (“Does the sentence make sense to the other person?”), but also shows up with general sentence sensibility judgements that do not directly call participants’ attention towards the comprehension of the social partner (“Does the sentence make sense?”)^10^. Crucially, these studies did not manipulate or require to infer the other’s belief states proper, therefore, it is not clear if the social N400 was elicited by mentalistic interpretive processes or more general social cognitive processes, such as detecting the informational asymmetry between the participant and the confederate. The key issue raised here is that inferential models of communication^1,2^ suggest that communicative intentions are attributed on the level of communicational pragmatics, which is preceded by processing “what was said”, based on syntactic-semantic computations. The N400, a marker of semantic comprehension, thus, may not be expected to be sensitive to mentalistic attributions of comprehension of meaning as intended, which is conceived to be computed afterwards, by pragmatic inferences.

Another approach to elicit the social N400 in infants^13,14^ was to induce a false belief in an Observer (instead of providing more or less semantic content). Objects were named always correctly for 14-month-olds, but labels sometimes became incongruent from the Observer’s perspective, because objects were replaced unbeknownst to her. Nevertheless, infants produced a social N400 effect, even though the labels were congruent with the objects in front of them. A notable import of this finding, beyond the observation that the social N400 may appear without manipulating the amount of semantic information, available to the participant and the Observer, is that the N400 seems to be sensitive to mentalistic manipulations.

What is common in the above two approaches is that an extended context is provided to participants, to which another person has limited access. Such a mismatch prompts participants to infer a meaning different from the one they reached. However, in the joint comprehension task^10,11^, there is a kind of information asymmetry between the referential context accessible to the participants and the confederate. Consequently, it is not clear in these paradigms whether actual mentalization takes place, or if there is simply a registration of the informational asymmetry of the context that induces the effect. In Forgács et al.’s^13^ paradigm, however, the extra context involves only beliefs. Therefore, belief attribution could be isolated from general social cognition in the context of semantic processing. (Notably, we focus specifically on semantic comprehension, not on language in general.) A key question arising from the above observations is the extent to which the social N400 is evoked simply by the presence of another person (and perhaps spontaneous mentalization), or due to the difference in perspectives of the other and the participant (which may induce the attribution of false beliefs). The present study aims to explore the nature of the social N400 by separating these two potential contributions.

The above results raise the possibility that the N400 effect is indicative of processes that are integral components of mentalization and not only of semantic processing per se. Neuroimaging studies further support this idea. Wernicke’s area, parts of the superior temporal sulcus (STS) and gyrus (STG) and the temporo-parietal junction (TPJ) are thought to be responsible for generating the N400 response^15–17^. Recent meta-analyses of mentalization also point to the left (and right) TPJ and STG for computing false beliefs in a domain general manner^18^. Language comprehension and the ability to establish consensual, common meaning during communication could have originated from and could function based on the neural structures dedicated to mentalization.

In the present paper we set out to investigate mentalistic aspects of establishing linguistic meaning, as reflected by the N400. Our main question is whether the social N400 indicates (A) that mentalization systems recruit language systems to generate semantic content that can be attributed to social partners; this possibility is consistent with prior, rather general social interpretations of the social N400. The language network^19^ may be recruited or influenced by social cognitive network(s)^18^, or both systems may be activated by a central processor. Another possibility (B) is that the social N400 indicates that semantic systems work in a profoundly mentalistic manner, that is, semantic content is computed based on the informative intentions of communicative partners: meaning is processed *as intended*. Under the first scenario, as the language system is making an attempt at anchoring meaning, it takes into consideration the mental state of partners to establish common ground^20^. A module, be it dedicated to mentalization^21^, to pragmatics^22^, or to pragmatics within mentalization^23^, could be activated in addition to the language module. The second scenario is that mentalistic and semantic computations are incorporated within a single system. Semantic content might not be computed bounded to the self or to the other, but to the group of interlocutors as a whole, whereby only the common ground is processed^14^, that is, the overlap between language as comprehended and comprehension as attributed to communicational partners. We have developed two approaches, where we use social presence and false beliefs as critical factors to identify the cognitive mechanisms of mentalization in communication of meaning. We manipulated systematically the elements of communicative situations to establish the minimal conditions for language comprehension and its attribution in a social context.

First, we have operationalized mentalization as a spontaneous process^24,25^ that can be prompted by the mere presence of a social partner. If the social N400 is sensitive to the presence of another person, it will be evidence that semantic and mentalistic systems are integrated. In contrast, if there are two separate systems for linguistic meaning and mentalization^14^, there should be no N400 effect, because there is no need to generate and attribute an additional, separate semantic representation (i.e. belief) to the other person.

Second, we have elicited false beliefs, a critical test of mindreading^26^, in a communicative situation. If a social N400, evoked via false beliefs, were accompanied by a frontal effect for mentalistic computations, as observed with infants^13,14^ and adults^27–29^, it would be evidence for two separate systems. If these systems are integrated, however, there should be no frontal effect, only an N400: the semantic system would generate meanings beyond the self, mentalistically, relevant in the communicative situation as a whole. This outcome would also suggest that observations with infants^13,14^ report of a developmentally earlier stage in mentalistic communication. In five alternative versions of two different experimental paradigms we aimed to separate the contributions of social and semantic cognitive systems to language comprehension processes.

## Results

### Experiment 1

In order to investigate the effect of spontaneous mentalization as elicited by the presence of another person on semantic comprehension, we measured event-related potentials (ERPs) over centroparietal electrode sites in a live setting. We labeled object correctly or incorrectly as in a typical N400 experiment, either in the presence, or in the absence of an Observer, in a blocked design. An Experimenter was seated behind a curtain on the right side of a puppet theater stage and placed objects on it, which were named accurately or inaccurately by playing back an audio recording. In the Social Presence block another experimenter, an Observer, was seated on the other side of the stage, in front of the participant, and visibly took notice of each object that appeared on the stage and that consequently labeled. In the Alone condition there was no Observer. We chose a 2 (Congruity vs. Incongruity) × 2 (Social Presence vs. Alone) blocked design to minimize a potential cross-contaminating effect of the presence of a social partner on participants being alone (as in a typical N400 experiment). All raw data for all experiments are available at https://osf.io/b2rze.

**Figure 1:**
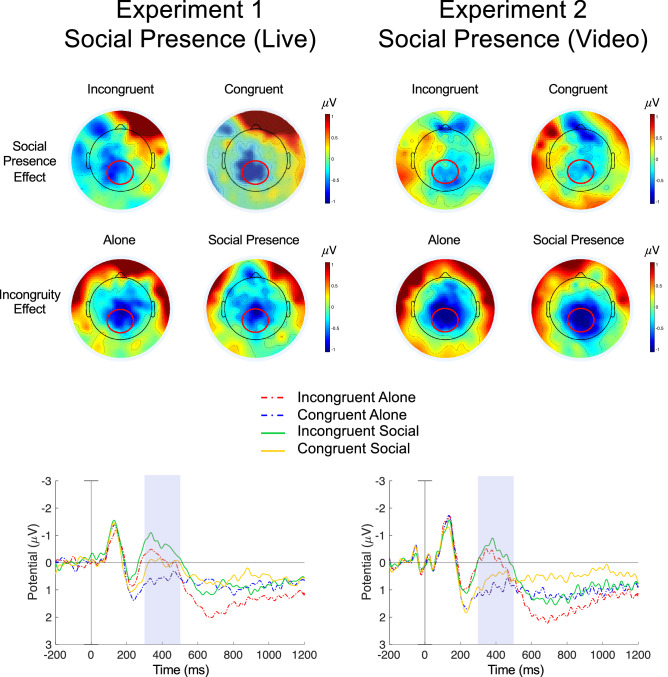
Electrophysiological responses observed in Experiment 1 & 2, evoked by semantic processing either in the presence or in the absence of a confederate. The left side shows results for the live presentation used in Experiment 1, and the right side shows result for the video presentation used in Experiment 2. Upper panels show topographical difference maps in the N400 time window (300–500 ms), where Social Presence Effect denotes a Social Presence–Absence difference, visualized for Congruent and Incongruent conditions separately; the Incongruity Effect denotes an Incongruent–Congruent difference, visualized separately within Social Presence and Alone conditions. Red circles mark a predefined frontal and centro-parietal Region-of-Interest (ROI). Lower panels show grand average ERP responses averaged over individuals and electrodes within each ROI. Time 0 is the onset of auditory word playback. Grey shades indicate N400 time windows where statistical analyses were carried out. Both a typical semantic Incongruity (N400) and a Social Presence effect, an enhanced electrophysiological negativity in the 300–500 ms time window over the centroparietal ROI are apparent in both experiments. The N400 response seems to be sensitive no only to semantic incongruities, but also to the mere presence of social partners. Perhaps it indicates the activation of a broader range of possible senses and meanings that the other person could have in mind, enabling rapid and fluent adjustments of the common ground, arriving at implied meanings and agreeing on what was meant by what was said. The finding suggests that the language system might be an integral part of mentalistic social-cognitive neural structures.

Thirty-four adults participated in this study. Results are shown in Fig. 1. We analyzed ERPs in the 300–500 ms time window over a centroparietal ROI, as usual for a typical N400^5^. A 2 × 2 ANOVA showed a significant main effect of Congruity, *F*(1, 33) = 17.18, *p* < 0.001, 90% CI [0.13 µV, 0.50 µV], η_*p*_^2^ = 0.40, and a significant main effect of Social Presence, *F*(1, 33) = 8.75, *p* = 0.006, 90% CI [0.04 µV, 0.39 µV], η_*p*_^2^ = 0.27, with no interaction, *F*(1, 33) = 0.04, *p* = 0.84, 90% CI [0.00 µV, 0.07 µV], η_*p*_^2^ = 0.001. Additional late effects were found frontally and parietally, which are reported (for all experiments) in Supplementary Results, Supplementary Fig. S1 and Supplementary Table S1. The main effect of an enhanced negativity evoked by social presence, especially with no interaction, was an unexpected and intriguing outcome of this experiment. It raises the possibility that the mere presence of another person could expand the range of activations in one’s semantic memory, irrespective of semantic congruity: possible alternative senses and meanings could be activated for congruent labels as well.

Importantly, in Experiment 1, participants were not entirely alone in the Alone condition: objects were placed on the puppet theater stage by an Experimenter, to whom participants could have attributed mental states. In order to exclude the possibility of such social contamination, we conducted a second experiment, in which we run the same paradigm but used recorded video clips that included only an single Observer in the Social Presence condition.

### Experiment 2

Experiment 2 was an exact video replica of the live presentation of Experiment 1. In the Alone condition, objects simply appeared on a stage (with no hand placing or removing them) and in the Social Presence block an Observer was visible in the video, facing the participant from the other side of the stage. We predicted the same outcomes we have observed in Experiment 1 and preregistered our video replication (https://osf.io/asmbj).

The results of 34 participants are shown in Fig. 1. The 2 × 2 ANOVA with Congruity and Social Presence as the two within-subject factors, carried out on the typical N400 yielded again no interaction (*p* = 0.97), but two significant main effects. There was a greater negativity for incongruent labeling, *F*(1, 33) = 38.4, *p* < 0.001, 90% CI [0.32 µV, 0.66 µV], η_*p*_^2^ = 0.67, and also to object naming in the Social Presence of another person, *F*(1, 33) = 5.05, *p* = 0.031, 90% CI [0.01 µV, 0.31 µV], η_*p*_^2^ = 0.22. We have replicated our previous finding: the N400 response appears to be sensitive to the mere presence of another person.

To better understand the effect of social partners on language comprehension beyond mere social presence, we have adapted Forgács et al.’s^13^ infant paradigm to adults, which requires mentalization in the form of attributing false beliefs—over and above the mere presence of an Observer.

### Experiment 3

**Figure 2:**
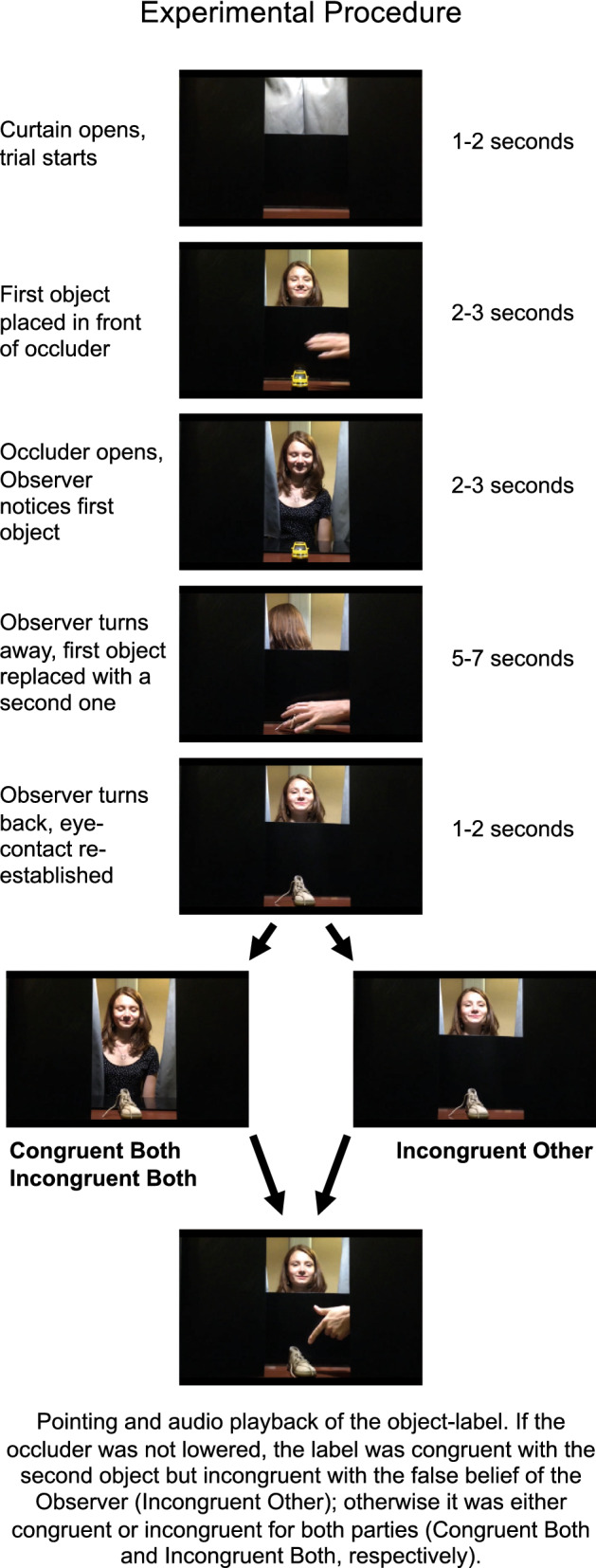
Experimental procedures of Experiments 3–5. The experimental paradigm was adapted from Forgács et al.’s studies^13,14^, and is described in detail in the Supplementary Procedures. In Incongruent-Other trials, due to an object change not visible for the Observer, the Observer held a false belief about the identity of the object being labeled, thereby she experienced a semantic incongruity. In the other two conditions the object change was revealed to the Observer by showing her the second object as well, and then objects were labeled either correctly (Congruent-Both) or incorrectly (Incongruent-Both). (Figure adapted from Forgács et al.^13^ with minor modifications to describe the present studies).

As a next step, we set out to study how mentalization, as measured via the attribution of false beliefs, influences semantic processing and the N400. False belief induction is considered to be a gold standard for investigating the attribution of mental states^26^. If the N400 is sensitive to information asymmetry between two interlocutors^10–12^, it could be expected to be sensitive to direct mentalistic manipulations as well. We implemented the live infant paradigm of Forgács et al.^13^ with adults. We placed an object (e.g., a toy car) on the puppet theater stage in front of participants, which was hidden from an Observer, who sat on the opposite side of the stage facing participants, by an occluder. The occluder was lowered, so that the Observer could identify the object, but then the occluder was raised, the Observer turned away, and the first object was replaced by a second object (e.g., a teddy bear) unbeknownst to the Observer. As the Observer turned back, if the occluder was lowered again, the Observer could update her representation concerning the object, but if the occluder remained in place, the Observer had a false belief regarding the identity of the object (Fig. 2). Then the object was pointed at and labeled, which was: either (1) a correct label, congruent for the participant but incongruent with the false belief of the Observer, because the occluder was not lowered and the label for the second object did not match the identity of the first object (Incongruent-Other); or (2) congruent for both parties, because the occluder was lowered and the object was labeled correctly (Congruent-Both); or (3) incongruent for both parties, because the occluder was lowered but the object was labeled incorrectly (Incongruent-Both). Additional details are available in the Supplementary Procedures. Just like in previous studies^10–12^, participants received instructions to follow the comprehension of the Observer and to mark on a response sheet whether the word label matched the object the Observer last seen. We predicted a social and a typical N400 in the same time window and over the same ROI as in Experiment 1 & 2.

**Figure 3:**
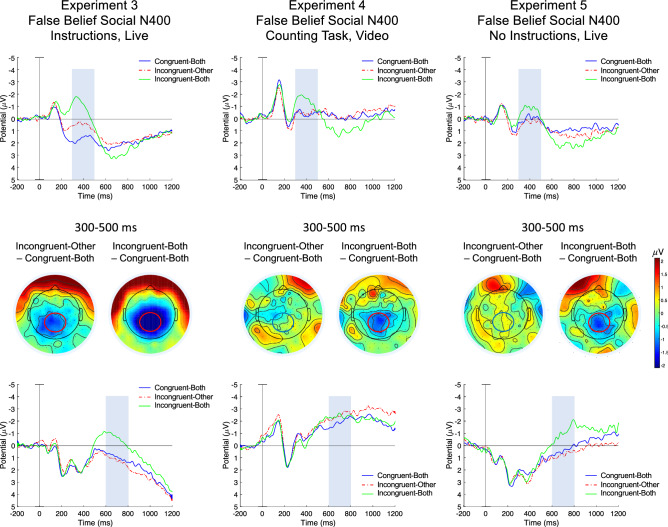
Electrophysiological responses observed in Experiments 3–5, evoked in a false belief social N400 paradigm with or without instructions. Top and bottom panels show grand average ERP responses over the centro-parietal and the frontal ROIs, respectively. Grey shades show time windows where statistical analyses were carried out (300–500 ms centroparietally and 600–800 ms frontally). Time 0 indicates audio word playback onset. Middle panels show topographical difference maps of indicated conditions in the 300-500 ms time window. A social N400 effect (Incongruent-Other–Congruent-Both) is apparent only when explicit instructions directed participants to mark on a response sheet if audio labels were correct from the perspective of an Observer (Experiment 3), but no such effect was evoked when there were no instructions to follow the language comprehension of the Observer (Experiment 4 & 5). Participants did not spontaneously follow the comprehension of a confederate seated facing them in a false belief situation, which suggests that interlocutors might not attend another person’s communicational perspective and mental state, unless explicitly required to do so. Additional late effects were observed and are reported in Supplementary Results (also see Supplementary Fig. S2).

Thirty-four participants took part in this study, their grand average ERPs are shown in Fig. 3. A one-way ANOVA with the within-subject factor of Condition revealed a significant effect of Condition, *F*(2, 66) = 24.8, *p* < 0.001, 90% CI [0.27 µV, 0.53 µV], η_*p*_^2^ = 0.43, with the Congruent-Both condition being less negative than both the Incongruent-Other (social N400), *t*(33) = 3.08, *p* = 0.004, 95% CI [0.37 µV, 1.83 µV], Hedges’ *g*_*av*_ = 0.56, and the Incongruent-Both conditions (typical N400), *t*(33) = 9.70, *p* < 0.001, 95% CI [2.01 µV, 3.08 µV], Hedges’ *g*_*av*_ = 1.22. We have observed a late frontal negativity in the 600–800 ms time window for Condition, *F*(2, 66) = 12.66, *p* < 0.001, 90% CI [0.12 µV, 0.40 µV], η_*p*_^2^ = 0.28, but pairwise comparisons revealed a difference only between the Incongruent-Both and Congruent-Both conditions, *t*(33) = 4.55, *p* < 0.001, 95% CI [0.81 µV, 2.13 µV], Hedges’ *g*_*av*_ = 0.55, not between the Incongruent-Other and Congruent-Both conditions (*p* = 0.55). Surprisingly, a late frontal effect accompanied not the social N400 but the typical N400, which is reported in the Supplementary Results (also see Supplementary Fig. S2 and Supplementary Table S2).

Since the social N400 was not evoked with no instructions in adults^10^, but it was apparent with infants^13^, the question arises whether instructions are necessary to elicit the social N400 response in adults using our false belief paradigm.

### Experiment 4

In order to investigate the role instructions play in the social N400 effect we run a video version of Experiment 3 where we did not ask participants explicitly to follow the comprehension of the Observer. In order to simplify the laborious live experimental procedure, we created a video recording of Experiment 3. To compensate for the attention grabbing effect of a physically present individual, we created a task that did not explicitly request belief tracking, but directed participants’ attention towards the visual perspective of the Observer. The task was to count how many times the Observer could have seen a particular object (e.g., a shoe) over the course of 10 trials, considering that the occluder is not always lowered. We predicted a social N400 effect and the same pattern of late frontal and parietal effects as in Experiment 3.

Seventeen adults participated in Experiment 4, the results are shown in Fig. 3. A one-way ANOVA indicated a significant effect of Condition, *F*(2, 32) = 3.87, *p* = 0.031, 90% CI [0.01 µV, 0.35 µV], η_*p*_^2^ = 0.20, and pairwise comparisons showed a typical N400 effect between Incongruent-Both and Congruent-Both conditions, *t*(16) = 2.31, *p* = 0.034 95% CI [0.09 µV, 2.04 µV], Hedges’ *g*_*av*_ = 0.57, but no social N400 effect between the Incongruent-Other and Congruent-Both conditions, *t*(16) = 0.22, *p* = 0.83, 95% CI [–0.75 µV, 0.92 µV], Hedges’ *g*_*av*_ = 0.05. Statistical analyses of the frontal negativity in the 600–800 ms time window showed no significant effect of Condition, *F*(2, 32) = 0.66, *p* = 0.52 (for more details see Supplementary Results).

Unexpectedly, we were not able to evoke a social N400 when we did note explicitly instruct participants to follow the comprehension of the Observer. We hypothesized that one possible explanation could be the video presentation format, not the lack of instructions. Prior infant and adult social N400 studies all employed live paradigms. Therefore, we created an exact, live replica of the infant paradigm^13^ with adults, where we provided no instructions.

### Experiment 5

Experiment 5 employed the exact same conditions as Experiment 4, but in a live, puppet theater setting, with no instructions. We aimed to exclude the possibility that the video presentation (and its monotony) produced the null-effect in Experiment 4, instead of the lack of instructions. More importantly, we sought to run an exact replication of the infant study with adults, therefore, we carried out the exact same procedures as Forgács et al.^13^, where no instructions were provided, except that we provide no instructions because it is a replication of an infant study.

Seventeen adults participated in Experiment 5. Results are shown in Fig. 3. A one-way ANOVA with Condition as the within-subject factor revealed a significant effect over the centroparietal ROI in the N400 time window, *F*(2, 32) = 8.47, *p* = 0.001, η_*p*_^2^ = 0.35, 90% CI [0.01, 0.35]. Pairwise comparisons showed a significant typical N400, *t*(16) = 3.18, *p* = 0.006, 95% CI [0.25 µV, 1.24 µV], Hedges’ *g*_*av*_ = 0.37, between the Incongruent-Both and Congruent-Both conditions, but no amplitude difference for the social N400, between the Incongruent-Other and Congruent-Both conditions, *t*(16) = –1.23, *p* = 0.24, 95% CI [–0.74 µV, 0.20 µV], Hedges’ *g*_*av*_ = 0.17. In the 600–800 ms time window over the frontal ROI, Condition was significant *F*(2, 32) = 6.03, *p* < 0.006, 90% CI [0.05 µV, 0.43 µV], η_*p*_^2^ = 0.27. The difference between the Incongruent-Both and Congruent-Both conditions was significant, *t*(1, 16) = 3.00, *p* < 0.008, 95% CI [0.44 µV, 2.56 µV], Hedges’ *g*_*av*_ = 0.79, but not between the Incongruent-Other and Congruent-Both conditions (*p* = 0.61). Thus, adults did not produce a social N400 effect in this paradigm either, and a late frontal negativity again accompanied only a typical N400.

Taken together, the results of Experiments 3–5 demonstrate that the social N400, when investigated in a false belief situation proper^13^, not by an information asymmetry paradigm^10–12^, can be evoked only using explicit instructions. There is a contrast between these two approaches because the social N400 was evoked in Jouravlev et al.’s^10^ study both with and without instructions to follow specifically the comprehension of a confederate, whereas in our false belief setting it showed up spontaneously in infants, but in adults only when they received explicit instructions to follow the comprehension of the Observer. Table 1 summarizes the predicted and obtained results of all five experiments.

**Table 1:** Summary of expected and observed results in all five experiments.

Experiment	Method	Expected results	Social Presence effect	False belief social N400 effect	Observed ERPs
Exp. 1. Social Presence 1	Live	Social Presence effect	✓	N/A	Enhanced N400
Exp. 2. Social Presence 2	Video	Social Presence effect	✓	N/A	Enhanced N400
Exp. 3. False Belief Social N400 1	Live, instructions	Social Presence effect + False Belief Social N400	✓	✓	Social N400
Exp. 4. False Belief Social N400 2	Video, counting task	Social Presence effect + False Belief Social N400	✓	X	No difference
Exp. 5. False Belief Social N400 3	Live, no instructions	Social Presence effect + False Belief Social N400	✓	X	No difference

## Discussion

We investigated the cognitive processes underlying the social N400 to answer in a novel way the question whether the social N400 reflect purely semantic processes or may indicate mentalistic computations involving the semantic system. We found that when linguistic comprehension could or should have been attributed to another person, the N400, traditionally associated with individual semantic comprehension, is sensitive to social presence and computes linguistic false beliefs in communicative situations. Specifically, the N400 response was evoked by experimental manipulations of mentalistic attributions of beliefs and language comprehension in paradigms that should not have influenced it. These results raise the possibility that the semantic system might be working in a profoundly mentalistic manner when it comes to inferring and anchoring linguistic meaning *as intended*.

The N400 response appeared to be sensitive not only to the comprehension of another person (Experiment 3), as reported previously as the social N400 effect^11^, but also to the mere presence of a social partner (Experiment 1 & 2). In other words, when listening to congruent and incongruent word labels alone, the N400 was reduced to a greater extent than if someone was present. In our false belief social N400 paradigm (Experiment 3), we recorded an additional N400 effect over and above the social presence effect: the response was not strongly reduced by a congruous label if another person falsely believed it was incongruous. Thereby we separated two apparently additive components of the social N400, both mentalistic in nature. Previous studies argued that adults spontaneously track the comprehension of others in information asymmetry paradigms^11,12^, however, using the adult adaptation of infant false belief procedures^13^, we demonstrated that attribution of semantic comprehension to a social partner is not spontaneous, because the false belief component of the social N400 was not evoked if participants were not explicitly instructed to track the comprehension of the other person who was present (Experiment 4 & 5).

The pattern of results implies that there are two mentalistic components to the neurocognitive computations responsible for the social N400, because it appears during social situations even if sentences or object labels are congruous on an individual level. In Experiment 1 & 2 we recorded an enhanced N400 response both to correct and incorrect object labels when another person was present, either physically or on a video screen. Based on the timing and overall centroparietal distribution of the effect, we suggest that there might be quantitative but no qualitative differences between the typical and the social presence N400. Even though the distribution was slightly skewed to the left, while the typical N400 is slightly skewed to the right^5^, the N400 is believed to be produced by multiple generators in the first place^15–17^. Since the typical N400 seems to reflect the activation of semantic memory systems^5,9^, the additional electrophysiological response evoked by social presence in the N400 time window could indicate an activation of a wider range of semantic elements, that is, broadening of meaning^30^. Additional, alternative meanings and word senses may be retrieved on behalf of, for and by the social partner to maintain the common ground^20^ and incorporate the cognitive environment^1^, the discourse, and the partner’s belief state.

Rueschemeyer et al.^11^ reported a social presence effect but only in terms of response accuracy, and an ERP study^31^ reported effects of social presence, a more centrally distributed left anterior negativity (LAN) for syntactic violations and a right anterior response for semantic violations, but not the modulation of the N400 proper. Just like in all prior studies, the confederate was seated next to the participant in the above experiment as well, but in our design the Observer was facing the participant, more akin to a communicative situation than the joint comprehension task, yielding high ecological validity.

We further investigated the influence of another person on language comprehension using a false belief paradigm (Experiment 3–5). Participants activated their neural systems responsible for the N400 to labels that were incongruent only for the Observer, not for them, and importantly, over and above a social presence N400 effect (an Observer was always present in these experiments). This false belief social N400 effect was smaller in magnitude relative to a typical N400 and appeared only following explicit instructions. Crucially, however, we observed no frontal responses reported previously during mentalization^27–29^. Therefore, the neurocognitive processes that give rise to the N400 response appear to incorporate mentalistic computations, specifically, the tracking of false beliefs, which may be responsible for identifying linguistic misunderstandings. The graded nature of the social N400, both within and across our studies, fits well with the graded N400 responses reported in literature. Not only social presence but the tracking of another person’s (mis)comprehension reduces the N400 to a lesser extent than comprehending alone, yet this neural mechanism has previously been associated primarily, and until recently exclusively, with individual semantic comprehension. We propose that the pattern of our results are inconsistent with models conceptualizing language and mentalization as separate modules but are consistent with the idea that language systems incorporate mind-reading mechanisms necessary for inferring meanings as intended by social partners.

In Experiments 4 & 5 we found that the social N400 effect disappears in lack of instructions to follow the comprehension of another person. This finding is in contrast with visual perspective taking experiments^24,25,32^ and suggests that belief representation requires deliberate effort^33,34^. In prior information asymmetry paradigms the spontaneous social N400 was evoked by different perspectives transmitted by language, not by false beliefs. It is possible that in lack of instructions our participants did not notice that they are in a false belief situation (carried away by the theatrical experimental setting). In short, mentalization could be composed of spontaneous elements induced by mere presence (linguistic perspective taking), and deliberate elements of mentalistic attributions (of false beliefs and comprehension), both of which appear to be integrated into the computation of meaning as reflected by the N400 response.

Evidence from neuroimaging provide further support for the idea that mentalistic and semantic systems may be integrated. Meta-analyses of neuroimaging studies on mentalization point to the TPJ bilaterally and the left STS as key hubs for computing mental contents for social partners^18^. Left TPJ and STS are thought to be responsible for generating the N400 as well^15,17,35^ and are core contributors to the semantic network^36^. Damage to these regions leads not only to aphasic symptoms but dramatically reduces the N400 response. The temporal pole, another contributor to the scalp N400^16^ responds to communicative intentions as well, such as being addressed and/or looked at^37^. An overlap of brain areas is no proof, but here we provide functional evidence that the N400 response is not merely influenced by general social cognition but evoked specifically by mentalization during language comprehension. Importantly, functional specificity of the language network is not established in contrast to social cognition let alone mentalization^19^. Semantic comprehension has mostly been studied in isolation (i.e. in written or audio formats), which could have restricted the scope of the investigations of the neural foundations of language and could have overlooked the mentalistic nature of semantic computations. An interesting novel framework^38^ proposes that TPJ and mentalization effects might reflect mental conflict monitoring rather than mental representations per se. On a first glance, such a view could prompt a reinterpretation of several findings, including our own, but it also seems to have its own challenges. First, it is difficult to see how it could account for social facilitation effects, such as altercentric processing advantage in perspective taking studies^32,39^. Second, in our experiments the Social Presence effect involves no mental conflict; the N400 maybe conceived as a “mismatch” or “violation” detector, but its graded nature^5^ suggests that, irrespective of its evoking conditions, it mostly reflects the state of the semantic memory system. Finally, in our false belief based social N400 paradigm only the Observer experienced a conflict. Even if a mental conflict explanation of prior mentalization findings prevails eventually, it still seems safe to assume that, for the basis of comparison, a mental model both for the self and for the other is computed. Therefore, our suggestion that the N400 is sensitive to the belief states of social partners should still hold.

Prior research on language processing was carried out primarily in individual experimental settings, where participants could have relied on a kind of “default” semantic processing. When an actual person is present, however, semantic activations might be generated for the specific partner and local common ground. Even though our experimental settings do not involve direct communication, they incorporate the minimal core conceptual assumptions of communication: semantic processing demands in the presence of another person. Anchoring word meaning in a social situations is a constitutive prerequisite for communication to take place. Differentiating between idealized vs. actual interlocutors puts our results in a broader pragmatic context, where linguistic inferences are made with respect to the particularities of the mental states of communicative partners. These findings corroborate inferential theories of communication^1,2^, where linguistic meaning is inferred based on mutual attributions of mental states, intentions and implications^30^. Meaning as intended by speakers are by and large underdetermined by word meaning^40^, and semantic processes might be mentalistic in this inferential sense: specific and exact word meanings may be inferred by hearers as implied and intended by speakers.

In sum, the N400 effect appears to be modulated by the mere presence of another person, perhaps due to the activation of a broader set of possible meanings attributed to her. It is further enhanced (or even less reduced) if another person has a misunderstanding because of a false belief, even if one’s own semantic expectations are not violated. Crucially, no previously reported (frontal) neural markers of mentalization accompany it. Our findings go far beyond the idea that social cognition in general influences language or communication. It is specifically mentalization that affects specifically semantic processing. Even though linguistic meaning and intentions seem to be inextricable intertwined, we sought to show the depth and nature of their interdependence. We suggest however, that semantics that is grounded in intentions, instead of social cognition simply influencing language use on a communicative-pragmatic level. These data together suggest that the semantic system may be a kind of mentalization system and that establishing intended meanings is a creative social process where meaning is built not only from the raw materials of speech and language but incorporates the mental contents attributed to social partners.

## Methods

### Participants

#### Sample sizes

We determined sample sizes based on effect size calculations and experience in our lab. We calculated the sample size of 34 participants based on the effect size reported by Rueschemeyer et al.^11^ (η_*p*_^2^ = 0.1 for the interaction) using G-Power^41^. We chose a single, larger sample size, required for the 1 × 3 repeated measures ANOVA (Experiments 3–5), and employed it for the 1 × 4 repeated measures ANOVAs (Experiments 1, 2) as well, for better comparability. In Experiments 4, 5 data collection was suspended at 17 participants after visual inspection indicated that no social N400 effect was evoked and data gathering would be redundant. No statistical tests were conducted prior to this decision. ERP effects should be visible already by around 5–10 participants and data gathering is regularly checked to confirm the correct functioning of the EEG system. Not simply visible, but significant N400^42^ and mentalization effects^27,29^ have been reported with 17 or less participants before.

Participants were always different in each study. All of them were native speakers of Hungarian, had normal or corrected to normal vision, clear hearing and reported no prior or acute neurological or psychiatric problems. Participants were either recruited from a course specifically designated for participation in experiments at Eötvös Loránd University (ELTE) for course credit, or through a student work organization and compensated for their participation (1500 HUF / hour). Written informed consent was obtained from all participants prior to the experiments.

#### Experiment 1

Thirty-four (24 female) healthy native speakers of Hungarian participated in Experiment 1 (age: *M* = 22.3 years, *SD* = 3.45, range 18–34). They were all right handed as measured by the Edinburgh Handedness Inventory (EHI)^43^ with a handedness index over 50 (*M* = 87.5, *SD* = 15). An additional eight participants were excluded due to noisy EEG data (blinks, eye-movements, and other EEG artefacts).

#### Experiment 2

Thirty-four participants (27 female) took part in this study (age: *M* = 22.1, *SD* = 2.84, range 18–32). They were right handed (EHI ≥ 75, *M* = 89.1, *SD* = 8.93). The study was preregistered at https://osf.io/asmbj. A further 19 participants were excluded due to noisy EEG data (apart from blinks, eye-movements and other typical EEG artefacts, some of the EEG nets gradually lost their measurement sensitivity due to aging, but this did not affect the quality of the included data, since all noisy trials were discarded).

#### Experiment 3

Thirty-four adults (27 female) participated in this study (age: *M* = 20.9, *SD* = 1.96, range 18–26). All were right handed (EHI ≥ 50, *M* = 82.4, *SD* = 16.0). An additional five participants were excluded due to noisy EEG data (blinks, eye-movements, and other EEG artefacts).

#### Experiment 4

Seventeen individuals (9 female) participated in this study (age *M* = 22.6, *SD* = 3.79, range 19–32). An additional two participants were excluded due to noisy EEG data (blinks, eye-movements, and other EEG artefacts).

#### Experiment 5

Seventeen participants (9 female) took part in this study (age *M* = 21.4, *SD* = 1.84, range 18–26). An additional six participants were excluded due to noisy EEG data (blinks, eye-movements, and other EEG artefacts).

### Materials

All five experiments involved 15 objects (an apple, ball, banana, book, bunny, car, cat, cup, dog, duck, phone, shoe, socks, spoon, and a teddy bear), adapted from Parise and Csibra’s study^42^ and the audio recordings of their labels, from Forgács et al.’s^14^. Objects were selected for an infant study that likely cover the vocabulary of 14-month-olds. The experiments utilized the same apparatus as Forgács et al.^14^: a puppet theater stage, with an occluder at is middle, operated by an Experimenter hiding behind a curtain. If the occluder was not lowered, objects remained hidden for the Observer, who was seated in front of participants on the other side of the stage. The Experimenter placed and replaced objects on the stage and the Observer played back audio recordings covertly using a button box. Trial instructions (sequence of objects and whether the occluder should be lowered or not) were presented for the Experimenter on a computer screen in front of her, on the left side of the stage, hidden from participants. We pseudo-randomized, using Python 3.6 (Python Software Foundation, Beaverton, Oregon, USA), the order of trials (so that the same condition did not appear more than twice in a row), of objects (so that the same object did not appear again in the next three trials) and of labels (so that no category member label was used in incongruent labelings); the experiment was controlled using Psychtoolbox running on Matlab (MathWorks Inc, Natick, Massachusetts, USA), including experimenter instructions, audio playback, and EEG triggering.

### Procedure

Ethics Committee of Eötvös Loránd University, Hungary approved our studies in advance, except for Experiment 4, for which approval was obtained from the United Ethical Review Committee for Research in Psychology (EPKEB) in Hungary. All experiments were performed in accordance with the relevant guidelines and regulations. In Experiment 1 participants were seated in a dimly lit room and watched a live puppet theater performance, while in Experiment 2 they watched a video recording of Experiment 1 in the same room. The sequence of events was the following. In the first block participants were seated alone in front of either a puppet theater stage (Experiment 1) or a screen (Experiment 2). In the live presentation of Experiment 1 an Experimenter, sitting hidden behind a curtain on the right side of the stage, placed objects on the stage, pointed at them, and played back an audio word label. Labels either matched to object or not (non-matching, incongruent labels were not of the same semantic category). After two seconds, the experimenter replaced the object with the next one. In the video presentation of Experiment 2 objects simply appeared on a table and were labeled either correctly or incorrectly. In block two, an Observer was seated in front of the participant, either physically (Experiment 1) or in the video (Experiment 2). Experiment 1 consisted of four blocks (45 trials each, thus 45 trials per condition) with no Observer in odd and an Observer present in even blocks. There were three short breaks between blocks, when participants could relax a bit and the EEG net could be readjusted as needed. Experiment 2 consisted of two blocks (90 trials each), the first with no Observer, the second with an Observer (again 45 trials per condition).

Experiment 3 and 5 were presented in a live performance, and Experiment 4 using a video recording of the exact same protocol. The protocol of these experiments was a combination of the two experiments reported by Forgács et al.^13^: objects were labeled either congruently or incongruently both for the participant and an Observer, but in the critical condition, labels were congruent only for the participant and incongruent for the Observer, who, due to a hidden object change, had a false belief about the identity of the object. In Experiment 3, participants’ task was to note on a response sheet their answer to the question: “Did the word label make sense for the Observer?”. In Experiment 4 the task was to count and mark on a response sheet how many times a particular object was seen by the Observer during the course of twelve trials (so that each of the 15 objects has to be tracked). There were no instructions in Experiment 5 apart from the explanation that it is a replication of an infant study, and just like infants, adults receive no verbal instructions. Experiment 3 and 5 consisted of four live blocks (30 trials each block, thus 45 trials per condition) and Experiment 4 was divided into two video blocks (72 trials each block, thus 48 trials per condition). Detailed experimental procedures are available in the Supplementary Procedures.

### EEG recording & analysis

EEG signal was recorded continuously with 128-channel Hydrocel Geodesic Sensor Nets at 500 Hz sampling rate using EGI’s Net Station 4.5.1. A 0.3 Hz high-pass and a 30 Hz low-pass filter was applied to raw data, which was segmented into epochs starting 200 ms before and lasting 1200 ms after the onset of the playback of the audio labels. Net Stations’ automatic artifact detection algorithms (for blinks, eye-movements, and extreme amplitudes) were employed to identify bad channels and segments, which was confirmed manually via visual inspection. A spherical spline interpolation was used to replace bad channels, a baseline correction was applied using a 200 ms window prior to audio onset, then segments were averaged for each condition, and finally re-referenced to the average reference. All raw EEG data are available at https://osf.io/b2rze. Based on prior research mean ERP amplitudes were calculated for the N400 in the 300–500 ms time window^5^ over a centroparietal Region-of-Interest (ROI) including 18 electrodes (31, 37, 53, 54, 55, 60, 61, 62, 67, 72, 77, 78, 79, 80, 85, 86, 87, REF); for the later frontal and parietal effects in the 600–800 time window^27,28^, over a frontal ROI as well with 18 electrodes (3, 4, 5, 6, 9, 10, 11, 12, 15, 16, 18, 19, 20, 22, 23, 24, 118, 124). Statistical calculations and reports follow Lakens’^44^ recommendations.

### Open practices statement

Experiment 1, 3, 4 and 5 were not formally preregistered; the preregistration for Experiment 2 can be accessed at https://osf.io/asmbj.

## Supplementary Information

Supplementary Information.

## Data Availability

Data for all experiments are posted at https://osf.io/b2rze. The materials used in these studies are widely available.

## References

[1] Sperber D, Wilson D (1986). Relevance: Communication and Cognition.

[2] Grice HP, Cole P, Morgan J (1975). Logic and conversation. Speech Acts.

[3] Kutas M, Hillyard SA (1980). Reading Senseless Sentences: Brain Potentials Reflect Semantic Incongruity. Science (80-).

[4] Kutas M, Federmeier KD (2000). Electrophysiology reveals semantic memory use in language comprehension. Trends Cogn. Sci..

[5] Kutas M, Federmeier KD (2011). Thirty years and counting: finding meaning in the N400 component of the event-related brain potential (ERP). Annu. Rev. Psychol..

[6] Van Berkum JJA, Hagoort P, Brown CM (1999). Semantic integration in sentences and discourse: Evidence from the N400. J. Cogn. Neurosci..

[7] Urbach TP, DeLong KA, Chan W-H, Kutas M (2020). An exploratory data analysis of word form prediction during word-by-word reading. Proc. Natl. Acad. Sci..

[8] DeLong KA, Urbach TP, Kutas M (2005). Probabilistic word pre-activation during language comprehension inferred from electrical brain activity. Nat. Neurosci..

[9] Brouwer H, Fitz H, Hoeks J (2012). Getting real about Semantic Illusions: Rethinking the functional role of the P600 in language comprehension. Brain Res..

[10] Jouravlev O (2019). Tracking Colisteners’ knowledge states during language comprehension. Psychol. Sci..

[11] Rueschemeyer SA, Gardner T, Stoner C (2015). The Social N400 effect: how the presence of other listeners affects language comprehension. Psychon. Bull. Rev..

[12] Westley A, Kohút Z, Rueschemeyer SA (2017). “I know something you don’t know”: Discourse and social context effects on the N400 in adolescents. J. Exp. Child Psychol..

[13] Forgács B (2019). Fourteen-month-old infants track the language comprehension of communicative partners. Dev. Sci..

[14] Forgács B (2020). Electrophysiological investigation of infants’ understanding of understanding. Dev. Cogn. Neurosci..

[15] Lau EF, Phillips C, Poeppel D (2008). A cortical network for semantics: (de)constructing the N400. Nat. Rev. Neurosci..

[16] Van Petten C, Luka BJ (2006). Neural localization of semantic context effects in electromagnetic and hemodynamic studies. Brain Lang..

[17] Federmeier KD, Laszlo S, Ross DH (2009). Time for meaning: Electrophysiology provides insights into the dynamics of representation and processing in semantic memory. Psychology of Learning and Motivation - Advances in Research and Theory.

[18] Schurz M (2020). Toward a hierarchical model of social cognition: A neuroimaging meta-analysis and integrative review of empathy and theory of mind. Psychol. Bull..

[19] Fedorenko E, Behr MK, Kanwisher N (2011). Functional specificity for high-level linguistic processing in the human brain. Proc. Natl. Acad. Sci. U. S. A..

[20] Clark, H. H. & Brennan, S. E. Grounding in communication. *Perspectives on socially shared cognition.* 127–149 (1991). 10.1037/10096-006.

[21] Leslie AM (1994). Pretending and believing: issues in the theory of ToMM. Cognition.

[22] Bosco FM, Tirassa M, Gabbatore I (2018). Why pragmatics and theory of mind do not (completely) overlap. Front. Psychol..

[23] Sperber D, Wilson D (2002). Pragmatics, modularity and mind-reading. Mind Lang..

[24] Elekes F, Varga M, Király I (2016). Evidence for spontaneous level-2 perspective taking in adults. Conscious. Cogn..

[25] Van Der Wel RPRD, Sebanz N, Knoblich G (2014). Do people automatically track others’ beliefs? Evidence from a continuous measure. Cognition.

[26] Dennett DC (1978). Beliefs about beliefs [P&W, SR&B]. Behav. Brain Sci..

[27] McCleery JP, Surtees ADR, Graham KA, Richards JE, Apperly IA (2011). The neural and cognitive time course of theory of mind. J. Neurosci..

[28] Liu D, Sabbagh MA, Gehring WJ, Wellman HM (2009). Neural correlates of children’s theory of mind development. Child Dev..

[29] Liu D, Sabbagh MA, Gehring WJ, Wellman HM (2004). Decoupling beliefs from reality in the brain: An ERP study of theory of mind. NeuroReport.

[30] Wilson, D. & Sperber, D. Truthfulness and relevance. In *Mind* vol. 111, 583–632 (Cambridge University Press, 2002).

[31] Hinchcliffe C (2020). Language comprehension in the social brain: Electrophysiological brain signals of social presence effects during syntactic and semantic sentence processing. Cortex.

[32] Freundlieb M, Kovács ÁM, Sebanz N (2018). Reading your mind while you are reading—evidence for spontaneous visuospatial perspective taking during a semantic categorization task. Psychol. Sci..

[33] Apperly IA, Butterfill SA (2009). Do humans have two systems to track beliefs and belief-like states?. Psychol. Rev..

[34] Schneider D, Lam R, Bayliss AP, Dux PE (2012). Cognitive load disrupts implicit theory-of-mind processing. Psychol. Sci..

[35] Kutas, M., Van Petten, C. K. & Kluender, R. Psycholinguistics Electrified II (1994–2005). *Handb. Psycholinguist.* 659–724 (2006). 10.1016/B978-012369374-7/50018-3

[36] Binder JR, Desai RH, Graves WW, Conant LL (2009). Where is the semantic system? A critical review and meta-analysis of 120 functional neuroimaging studies. Cereb. Cortex.

[37] Kampe KKW, Frith CD, Frith U (2003). ‘Hey John’: Signals conveying communicative intention toward the self activate brain regions associated with ‘mentalizing’, regardless of modality. J. Neurosci..

[38] Deschrijver E, Palmer C (2020). Reframing social cognition: Relational versus representational mentalizing. Psychol. Bull..

[39] Kovács ÁM, Teglás E, Endress AD (2010). The social sense: Susceptibility to others’ beliefs in human infants and adults. Science (80-).

[40] Wilson D, Carston R, Burton-Roberst N (2007). A unitary approach to lexical pragmatics: Relevance, inference and ad hoc concepts. Pragmatics.

[41] Faul F, Erdfelder E, Lang A-G, Buchner A (2007). G*Power 3: A flexible statistical power analysis program for the social, behavioral, and biomedical sciences. Behav. Res. Methods.

[42] Parise E, Csibra G (2012). Electrophysiological evidence for the understanding of maternal speech by 9-month-old infants. Psychol. Sci..

[43] Oldfield RC (1971). The assessment and analysis of handedness: The Edinburgh inventory. Neuropsychologia.

[44] Lakens D (2013). Calculating and reporting effect sizes to facilitate cumulative science: a practical primer for t-tests and ANOVAs. Front. Psychol..

