# Supplementary material for: Semantic systems are mentalistically activated for and by social partners
Source: Sci Rep. 2022 Mar 22;12:4866. doi: 10.1038/s41598-022-08306-w (PMC8941134; doi:10.1038/s41598-022-08306-w)
Supplement: Supplementary file 1 — Supplementary Information. [file 41598_2022_8306_MOESM1_ESM.pdf]

Supplementary Information

Semantic systems are mentalistically activated for and by social partners

Bálint Forgács, Judit Gervain, Eugenio Parise, György Gergely, Lívia Priyanka Elek, Zsuzsanna Üllei-Kovács, & Ildikó Király

Corresponding author: Bálint Forgács

Email: forgacs.balint@ppk.elte.hu

Website: https://sites.google.com/view/balint-forgacs

Supplementary Results

Late frontal and parietal effects. The late effects reported below do not contribute strongly to the main arguments of our paper, but for the sake transparency and clarity, we report them in their entirety.

Experiment 1. We have observed two additional effects following the N400: a P600-like late parietal positivity and a late frontal negativity as well. Exploratory statistical analysis of ERP responses over centroparietal regions in the 600-800 ms time window yielded a main effect of Congruity, $F(1, 33) = 16.7$, $p < .001$, 90% CI [0.12 μV, 0.50 μV], $\eta_p^2 = 0.32$, and of Social Presence, $F(1, 33) = 4.19$, $p = .049$, 90% CI [0.0004 μV, 0.28 μV], $\eta_p^2 = 0.21$, together with an interaction of the two, $F(1, 33) = 6.23$, $p = .018$, 90% CI [0.02 μV, 0.34 μV], $\eta_p^2 = 0.16$. When the interaction was broken down by Social Presence, the late parietal positivity was apparent only in the Alone condition, $t(33) = –4.52$, $p < .001$, 95% CI [–1.43 μV, –0.54 μV], Hedges' $g_{av} = 0.71$, not in the Presence ($p = .29$). The frontal negative response also showed up in the 600-800 ms time window, as a single Congruity main effect, $F(1, 33) = 10.7$, $p = .003$, 90% CI [0.06 μV, 0.42 μV], $\eta_p^2 = 0.29$ (other $p > .09$). Interestingly, these two late effects were showed up in a simple object naming paradigm where only an N400 was expected. The late parietal positivity appeared in the Alone condition when the label was incongruent, but disappeared in the Presence of another person, while the frontal negativity accompanied the N400 response irrespective of social Presence.

Experiment 2. The late parietal positivity showed a somewhat different pattern as before: there was no interaction ($p = .98$), but two significant main effects of Congruity, $F(1, 33) = 2.54$, $p < .001$, 90% CI [0.21 μV, 0.58 μV], $\eta_p^2 = 0.46$, and of Social Presence, $F(1, 33) = 1.21$, $p = .001$, 90% CI [0.07 μV, 0.44 μV], $\eta_p^2 = 0.34$. The late parietal positivity effectively lined up with the N400 response this time: there was a greater positivity following a typical N400 and also in the presence of another person. The analysis of the late frontal negativity brought a significant main effect of Congruity, $F(1, 33) = 33.5$, $p < .001$, 90% CI [0.29 μV, 0.63 μV], $\eta_p^2 = 0.47$, no effect of Social Presence ($p = .21$), but an interaction of the two, $F(1, 33) = 9.85$, $p = .004$, 90% CI [0.05 μV, 0.41 μV], $\eta_p^2 = 0.23$. When the interaction was broken down by Social Presence, a late frontal negativity was apparent when participants heard incongruent object labels Alone, $t(33) = 2.9$, $p = .007$, 95% CI [0.22 μV, 1.27 μV], Hedges' $g_{av} = 0.46$, but only marginally significant when in the Presence of an Observer $t(33) = 1.98$, $p = .056$, 95% CI [–0.02 μV, 1.02 μV], Hedges' $g_{av} = 0.28$. The late results of Experiment 2 diverge from those Experiment 1: the P600 effect mirrored the N400 response (in Experiment 1 it was evoked by incongruent labels only when participants were alone), while the frontal response was elicited by incongruent labels only when alone (in Experiment 1 it was sensitive to congruity regardless of social presence). The results of Experiment 1-2 are summarized in Supplementary Figure S1 and Supplementary Table S1.

Experiment 3. We did not observe a late centroparietal positivity, the effect of Condition was not significant, $F(2, 66) = 2.24$, $p = .12$ (only a late frontal negativity, between the Congruent-Both and Incongruent-Both conditions that we report in the main text).

Experiment 4. Statistical analyses revealed a significant parietal positivity over the centroparietal ROI in the 600-800 ms time window for Condition, $F(2, 32) = 5.79$, $p = .007$, 90% CI [0.05 μV, 0.42 μV], $\eta_p^2 = 0.27$. Pairwise comparisons showed a significant difference for the participant, between the Congruent-Both and Incongruent-Both conditions, $t(16) = -2.68$, $p = .017$, 95% CI [–2.18 μV, –0.25 μV], Hedges' $g_{av} = 0.79$, but not for the other, between the Incongruent-Other and Incongruent-Both conditions ($p = .99$). Statistical analysis of the frontal negativity over the frontal ROI in the 600-800 ms time window showed a non-significant effect of Condition, $F(2, 32) = 0.66$, $p = .52$. Visual inspection of the data indicated that there might be an effect between 500-700 ms, but exploratory statistical analyses (against a Bonferroni corrected $\alpha = .025$) revealed no significant effect of Condition in this time window either, $F(2, 32) = 1.026$, $p = .37$.

Experiment 5. A one-way ANOVA of ERPs over the centroparietal ROI in the 600-800 ms time window showed an effect for Condition $F(2, 32) = 5.79$, $p = .007$, 90% CI [0.05 μV, 0.42 μV], $\eta_p^2 = 0.27$, with a significant effect only for the self, between the Congruent-Both and Incongruent-Both conditions, $t(16) = -2.96$, $p = .009$, 95% CI [–1.99 μV, 0.33 μV], Hedges' $g_{av} = 0.53$, but not for the other, between the Incongruent-Other and Incongruent-Both conditions ($p = .23$). In the 600-800 ms time window over the frontal ROI Condition was significant $F(2, 32) = 6.03$, $p < .006$, 90% CI [0.05 μV, 0.43 μV], $\eta_p^2 = 0.27$. The difference for the self between the Congruent-Both and Incongruent-Both conditions was significant, $t(1, 16) = 3.00$, $p < .008$, 95% CI [0.44 μV, 2.56 μV], Hedges' $g_{av} = 0.79$, but not for the other, between the Incongruent-Other and Incongruent-Both Incongruent-Other and Incongruent-Both conditions ($p = .61$). In sum, we found an initially unpredicted yet consistent combination of late effects mostly in typical N400 contrast, which were insensitive to mentalization. The results of Experiment 3-5 are summed up in Supplementary Table S2 and Supplementary Figure S2.

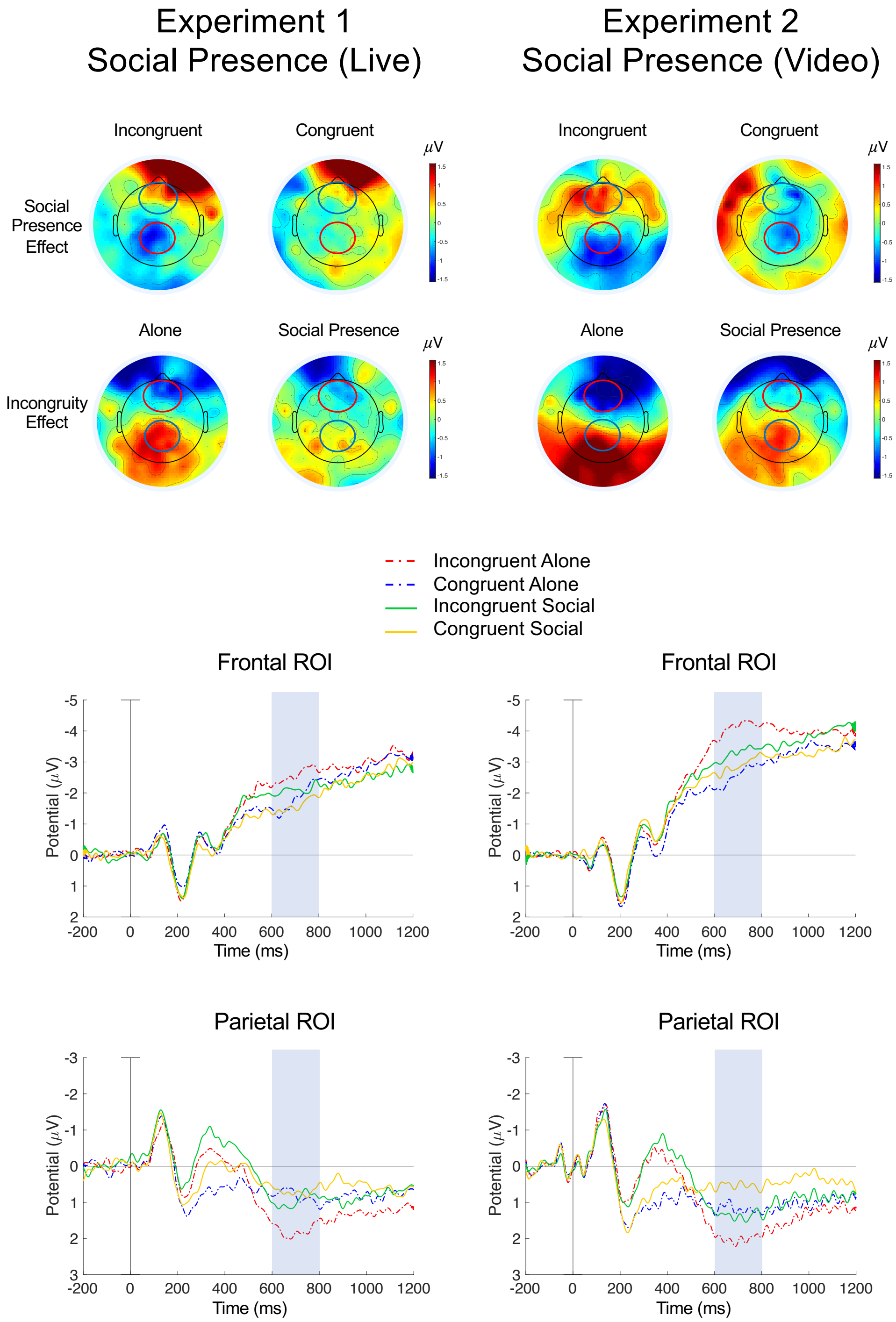

Supplementary Figure S1. In the Social Presence Experiment 1-2, late frontal and parietal effects appeared in a typical semantic incongruity situation. In the live paradigm of Experiment 1, a late frontal effect accompanied an N400 during semantically incongruous object labeling, irrespective of Social Presence. A P600-like late parietal effect showed up only in the Alone condition, not in the Social Presence condition. In contrast, in the video paradigm of Experiment 2, a late frontal effect appeared only in the Alone condition, while the P600-like response was accompanied the N400, as an additional Incongruity effect, but it was also sensitive to Social Presence: it was more positive when participants were Alone.

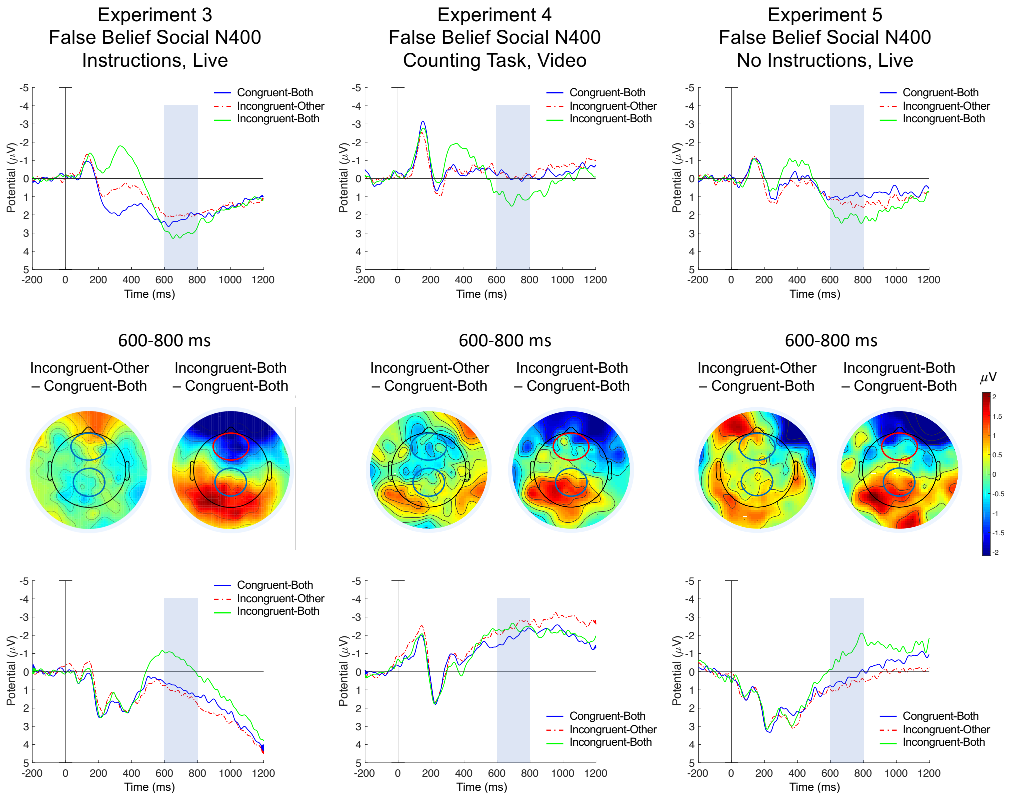

Supplementary Figure S2. The late frontal and parietal effects are an intriguing, unexpected but consistent findings of Experiment 3-5 as well, which we do not have clear explanation for. In all of our experiments they appeared in conditions where only a typical N400 was expected, perhaps indicative of preparing for or making an effort to correct an apparent and/or potential breakdown in common ground or in communication in general. Even though no false belief attribution was necessary in such contrasts in Experiment 3-5, mentalization processes could have been triggered by a potential interruption of the common ground [1,2] triggered by incorrect labels. Both (temporo-)parietal and frontal responses have been linked with mentalization processes in perspective taking [3] and false belief paradigms [4,5]. A late positivity or, as sometimes labelled in psycholinguistic research, a P600 effect, has been hypothesized to indicate high level semantic integration [6–8]. In our studies it could have indicated an attempt to integrate the object, the label, and the mental state attributed to the Observer. One possible explanation is that such effects work similarly to the N400: they are always elicited but are reduced when sufficient and relevant information is available. The late parietal positivity appeared only when alone in Experiment 1 just as well as the frontal negativity in Experiment 2, thus it might be reduced when linguistic input is easy to integrate with the social-pragmatic aspects of the communicative situation. For example, a P600-like effect is reduced when a bilingual confederate could have understood a target sentence that a monolingual could have not [9]. Likewise, the late frontal negativity might be reduced when mental state attribution is fluent, appears straight forward, and mentalization is not obstructed by conflicts of perspectives or intentions. Further studies are necessary to clarify the role of these late effects in language comprehension.

Supplementary Table S1. Pattern of results in Experiment 1-2, exploring the effect of Social Presence on semantic processing in an object naming paradigm, either in the presence of absence of a social partner.

	N400 response		P600 response		Frontal response	
	Incongruity Effect	Social Presence Effect	Incongruity Effect	Social Presence Effect	Incongruity Effect	Social Presence Effect
Experiment 1 Social Presence N400 Live	✓	✓	Interaction:	Alone only	✓	
Experiment 2 Social Presence N400 Video	✓	✓	✓	✓	Interaction:	Alone only

Supplementary Table S2. Pattern of results for Experiment 3-5 investigating the social N400 in an object naming false belief paradigm.

	N400 Effect		P600 Effect		Frontal Effect	
	Self	Other	Self	Other	Self	Other
Experiment 3 Social N400 Instructions	✓	✓			✓	
Experiment 4 Social N400 Video	✓		✓			
Experiment 5 Social N400 Live	✓		✓		✓	

Supplementary Methods

Supplementary Procedures. In Experiments 3-5 each trial was carried out in the following way. First, the Observer, who was seated on the other side of the puppet theater stage, opened a curtain right in front of her, and established eye-contact. Then, an object (e.g., a bunny) was placed in front of a mobile occluder in the middle of the stage of the puppet theater by an Experimenter, who was seated on the right side of the stage, hiding behind a curtain. Next, the occluder was lowered by the Experimenter, so that the object was revealed to the Observer – and she visibly looked at it and ensured that the participant saw that she saw the object. The occluder was raised, the Observer turned away 90° to her right, and without having visual access to it, the object was replace by another object (e.g., toy car) by the Experimenter. Then, the Observer turned back, and from this point there were three possible scenarios. In the Congruent-for-Both condition the occluder was lowered again, the Observer noticed the second object (an updated her false belief), the occluder was raised again, the Experimenter pointed at the object and the label of the object (e.g., "car") was played back from an audio recording (in fact by the Observer, secretively, to ensure that participants did not blink or move during playback). In the Incongruent-for-Both condition the second object was revealed just as well, but this time the audio playback was the label of a third object (e.g., "spoon"), thus incongruent for both parties. In the critical, Incongruent-for-Other condition, however, the second object was not revealed to the Observer: after she turned back to face the participant, the occluder was not lowered, and the Experimenter immediately pointed at the object and its label was played back from an audio recording (e.g., "car"), which was incongruent with the false belief of the Observer (e.g., *bunny*), while it was congruent with the object that was clearly visible for the participant. After a 1-2s the Experimenter removed the object, the Observer closed the curtain, and following an 1-2s break the next trial begun with the Observer opening the curtain again. The task of participants was to mark on a response sheet, whether the object label was correct for (i.e. from the perspective of) the Observer.

In Experiment 4 the protocol was video recorded and played back on a screen. A coy task was assigned for participants: they had to count how many times the Observer could have seen a particular object (e.g., a teddy bear) during a period of 15 trials. This way attention was slightly directed towards the perspective of the Observer, but no explicit instructions were given to follow her comprehension. Experiment 5 was again a live performance, but this time no instructions were provided, whatsoever, except that participants were explained that it is the adult version of an infant study, and as we are not able to provide instructions for infants, we do not provide instructions for them either. Additionally, prior to the experimental procedure, participants were invited to take the seat of the Observer, and it was explained to them that the occluder is not transparent and that objects come in a computer generated random order, so the Observer has no idea what object is place on the stage unless the occluder is lowered. Participants were requested to try not to blink (or blink less) when they saw a pointing hand.

Supplementary References

1. Clark, H. H., Schreuder, R. & Buttrick, S. Common ground at the understanding of demonstrative reference. *J. Verbal Learning Verbal Behav.* **22**, 245–258 (1983).
2. Apperly, I. Mindreading and Psycholinguistic Approaches to Perspective Taking: Establishing Common Ground. *Top. Cogn. Sci.* **10**, 133–139 (2018).
3. McCleery, J. P., Surtees, A. D. R., Graham, K. A., Richards, J. E. & Apperly, I. A. The neural and cognitive time course of theory of mind. *J. Neurosci.* **31**, 12849–12854 (2011).
4. Liu, D., Sabbagh, M. A., Gehring, W. J. & Wellman, H. M. Decoupling beliefs from reality in the brain: An ERP study of theory of mind. *Neuroreport* **15**, 991–995 (2004).
5. Liu, D., Sabbagh, M. A., Gehring, W. J. & Wellman, H. M. Neural correlates of children's theory of mind development. *Child Dev.* **80**, 318–326 (2009).
6. Brouwer, H., Fitz, H. & Hoeks, J. Getting real about Semantic Illusions: Rethinking the functional role of the P600 in language comprehension. *Brain Res.* **1446**, 127–143 (2012).
7. Delogu, F., Brouwer, H. & Crocker, M. W. Event-related potentials index lexical retrieval (N400) and integration (P600) during language comprehension. *Brain Cogn.* **135**, 103569 (2019).
8. Sassenhagen, J., Schlesewsky, M. & Bornkessel-Schlesewsky, I. The P600-as-P3 hypothesis revisited: Single-trial analyses reveal that the late EEG positivity following linguistically deviant material is reaction time aligned. *Brain Lang.* **137**, 29–39 (2014).
9. Kaan, E., Kheder, S., Kreidler, A., Tomić, A. & Valdés Kroff, J. R. Processing Code-Switches in the Presence of Others: An ERP Study. *Front. Psychol.* **11**, 1–18 (2020).
